# Systematic Review: Microfluidics and Plasmodium

**DOI:** 10.3390/mi12101245

**Published:** 2021-10-14

**Authors:** Nicolas Thorne, Luis Flores-Olazo, Rocío Egoávil-Espejo, Emir A. Vela, Julien Noel, Julio Valdivia-Silva, Danny van Noort

**Affiliations:** 1 Centro de Investigación en Bioingeniería, Universidad de Ingenieria y Tecnologia (UTEC), 15063 Lima, Peru; lflores@utec.edu.pe (L.F.-O.); regoavile@gmail.com (R.E.-E.); evela@utec.edu.pe (E.A.V.); jnoel@utec.edu.pe (J.N.); jvaldivias@utec.edu.pe (J.V.-S.); 2 Department of Mechanical Engineering, Universidad de Ingenieria y Tecnologia (UTEC), 15063 Lima, Peru; 3 Biotechnology, Linköping University, 581 83 Linköping, Sweden

**Keywords:** plasmodium, microfluidics, lab on a chip, Peru

## Abstract

Malaria affects 228 million people worldwide each year, causing severe disease and worsening the conditions of already vulnerable populations. In this review, we explore how malaria has been detected in the past and how it can be detected in the future. Our primary focus is on finding new directions for low-cost diagnostic methods that unspecialized personnel can apply in situ. Through this review, we show that microfluidic devices can help pre-concentrate samples of blood infected with malaria to facilitate the diagnosis. Importantly, these devices can be made cheaply and be readily deployed in remote locations.

## 1. Introduction

In 2018, there were around 228 million cases of malaria worldwide, of which 405,000 were fatal, with most of them in African countries (93%) [1]. The most vulnerable population are pregnant women (affecting fetus health, and leading to prematurity, low birthweight, and finally neonatal and infant mortality), and children [2]. While most countries recorded a decline in cases, the number of cases in the Americans is rising. Besides the human toll, malaria also influences the economy of countries with high rates of infection, up to 2% of the gross domestic product (GDP) [3], which can lead to poverty of the affected families [4,5]. This review focuses on malaria’s current diagnostic methods and their applications in underdeveloped countries, such as Peru.

Malaria is caused by a protozoan parasite called *Plasmodium*. Four different species of *Plasmodium* cause human infections: *P. falciparum*, *P. malariae*, *P. ovale* and *P. vivax*. Worldwide, the most prevalent species is *P. falciparum* (around 96%), which is the most lethal species (300,000 deaths worldwide in 2017 [6,7]), and *P. vivax*, which has the most extensive geographical distribution [8,9]. Occasionally, humans can get infected with a fifth species that typically infects animals: *P. knowlesi*.

In contrast with other pathogens, *Plasmodium* efficiently infects the host by entering cells. In mammalian hosts, they are transmitted by the bite of the female Anopheles mosquito infected with *Plasmodium* spp., which it houses in its salivary glands as sporozoites. When a mosquito feeds, the sporozites are injected into the hosts’ bloodstream [10,11,12]. Another possible source of infection is blood transfusions.

**Figure 1:**
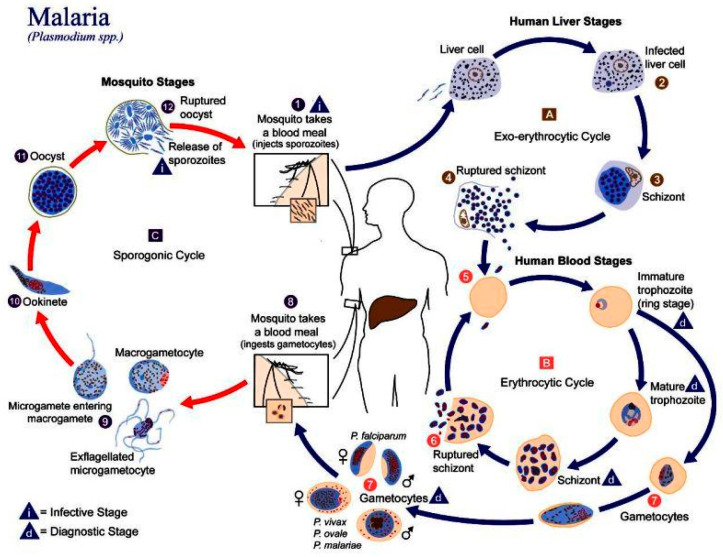
The life-cycle of *Plasmodium* spp. Reprinted, with permission, from the Centers for Disease Control and Prevention website, https://www.cdc.gov/malaria/about/biology/, accessed on 3 October 2021. Use of this material, does not imply endorsement or recommendation by CDC, ATSDR, HHS or the United States Government of this research. Copyright, 2020, Centers for Disease Control and Prevention.

In humans, the parasite has two cycles: exoerythrocytic and erythrocytic (Figure 1). During the exoerythrocytic cycle, *Plasmodium* spp. matures to the hypnozoite form and infects hepatocytes to become hepatic schizonts; these can remain in the liver for many years [13]. The parasite breaks through hepatocytes at the merozoite stage to infect red blood cells (RBC). It develops into early trophozoites (ring form), late trophozoites, and finally into erythrocytic schizonts. They can also turn into gametocytes and can then be suctioned by another *Anopheles* spp., thus repeating the cycle [14]. *Plasmodium* adheres to the RBC via a high-affinity ligand–receptor interaction from where it will invade the cell. An infection will cause extensive changes to the RBC, such as a change in cell morphology, increased rigidity, elevated permeability, and changes in iron content [15]. These changes in the RBCs can lead to cerebral malaria or anemia.

## 2. Malaria in Peru

Malaria has been a health issue in Peru since colonial times and is thought to have arrived with the migration of slaves from Africa. In the 17th century, the discovery of quinine from the endemic tree of Peru Cinchona officinalis was decisive for treating patients with this disease. Ever since, malaria has been a primary national health concern in Peru [16,17].

Epidemiologically, Peru follows the trend of the American Region, with *P. vivax* being the predominant species in the country (80.2% of the total cases), followed by *P. falciparum* (19.8%); the provinces with the most cases are Loreto, Amazonas, and Junin (95.8% of cases in the country) [18]. The lack of access to health centers and the fact that malaria shares some symptoms with other endemic diseases make the diagnosis more difficult and time-demanding, enabling the progression of the disease. It is calculated that the treatment of malaria costs a third of the family income of each patient [4,5]. To control the disease, different administrations have created policies to address different aspects of the problem: epidemiological control, prevention programs, provision of different medicines according to epidemiological characteristics, the control of *Anopheles* spp., and the application and design of different screening methods [17]. Since 2018, Peru has incorporated the Malaria Zero Program, a community-focused approach, and attempts to eradicate the disease by 2030. Unfortunately, with the outbreak of COVID-19, the activities of this program have been severely affected, primarily epidemiological surveillance, patient treatment, research projects, and educational activities in communities [5].

The most used diagnostic method in Peru is the Giemsa stain. Although its precision and low cost make it a powerful method, its reliance on infrastructure and specialists makes diagnostics with this technique challenging in poor and remote areas [4]. Another popular diagnostic method is PCR; its popularity is due to its high precision and throughput. However, the cost of PCR tests makes them prohibitively expensive for most of the country. That is why we, as a research group in Peru, are looking for a cheaper and faster way to perform diagnostics.

## 3. Current Methods of Diagnostics

Fast diagnosis and prompt treatment are necessary to prevent mild cases of Malaria from turning more serious, which could result in death. Presently, most diagnoses are performed with either optical means, which requires a microscope, or so-called rapid diagnostic tests (RDTs). The most commonly used optical method is the blood smear, which determines the presence of malaria parasites in the blood cells [19]. However, a low concentration of the malaria parasite can result in false negatives, as there might not be a parasite in the sample taken and imaged under the microscope [20]. The detection threshold under field conditions is estimated to be 50–100 parasites/mL [21,22]. In a laboratory setting, this threshold is 4–20 parasites/mL [23]. There can also be errors in species identification. Failures to differentiate *P. falciparum* from *P. vivax*, the two most common species of malaria, are frequent in routine microscopic diagnostics, and are therefore under-reported [22].

The same can be said for RDTs. RDTs rely on antigen–antibody interaction, resulting in a visible band on a paper strip, in the same manner as pregnancy tests. However, if there is a low concentration of malaria parasites in the blood, no band will show, leading to a negative result. The other drawback is that RDT does not screen for multiple strains of the parasite. Another promising POC method to detect malaria is loop-mediated isothermal amplification (LAMP). This method is able to detect quantities of the parasite that are below the detection threshold for other methods. LAMP has been shown to achieve specificities of up to 100% and sensitivities up to 98% [24].

Other methods, such as DNA probes and PCR [25], fluorescent staining using flow cytometry [26], detection of malaria pigments by depolarized laser light [27], and mass spectrometry [28], are either not suitable for point-of-care (POCs) in remote locations or show limited success.

Therefore, better, i.e., more sensitive, differential, and faster, POC diagnostics methods are required, which can be used in remote locations when necessary. Microfluidics can play an important role in such devices. This review will give an overview of existing microfluidic-based devices that can be used for either the detection, separation, or diagnosis. At the end of the review, we will also suggest other devices envisioned to help combat malaria on all levels.

## 4. Paper-Based Microfluidics and Malaria

**Figure 2:**
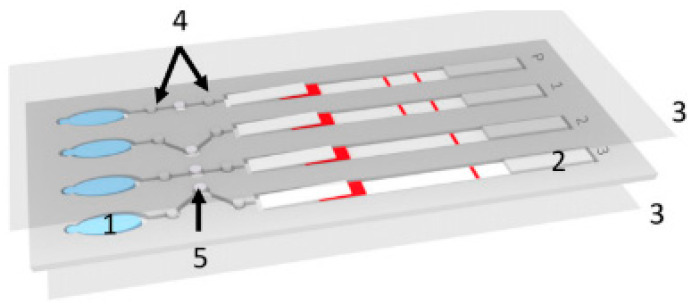
Example of a lateral flow device from Reboud et al. (1) buffer chamber which acts as a pump when pressed. (2) Lateral flow detection strip. (3) Acetate films to cover the device. (4) Filter valves made from paper to prevent the reagents from mixing prematurely. (5) Filter paper for LAMP reaction. Reprinted with permission from. © 2018 National Academy of Sciences.

One method that is suitable for use in remote locations because of its low cost and ease of operation is paper-based microfluidics, otherwise known as lateral flow devices [28]. Because of the properties of paper, these devices use capillary forces to pump fluids laterally through the paper, an example of this can be seen in Figure 2. Here, a paper lateral flow device (item 2) is used to perform a multistep assay [29]. Advances in paper-based microfluidic analysis have addressed some of the limitations previously associated with lateral flow devices, such as the inability to create valves [30] and multiplex or perform multi-step analysis [31]. Paper-based lateral flow devices have been primarily used to automate multistep assays quickly and cheaply. For malaria detection, the literature shows that loop-mediated isothermal amplification (LAMP) and signal-amplified sandwich format immunoassay for PfHRP2 are popular methods of detecting the presence of an infection. The results from these methods have been shown to be comparable to those obtained using ELISA [32].

Implementation of improved signal amplification techniques has increased the reliability of the readout [33]. Some of these improvements have been applied to detecting malaria in whole and pretreated blood, such as the detection of specific proteins in a blood sample. Fu et al. used a 2D paper network to filter whole blood and deliver appropriate volumes of the sample mixed with antibodies, rinse buffer, and a signal amplification reagent [32]. The device consists of a horizontal channel with a large pad (reaction area) at the end. The channel has three side channels at different positions down the main channel. The distance between the side channels and the reaction area determines the time it takes for the reagent to flow through the side channel to reach the reaction pad. With this device, Fu et al. were able to identify fetal bovine serum (FBS) spiked with different amounts of *Plasmodium falciparum* histidine rich protein PfHRP2. They showed that this technique is very promising as it is easy to use and can store the reagents on the device in a dehydrated form.

Another method to control the timing of reactions in a paper microfluidic device is by using microfluidic actuators (sponges), which expand at a known rate [34]. Toley et al. used this method to perform a similar process as Hu et al. and successfully detected PfHRP2 in a sample.

Other paper-microfluidic devices can process pretreated blood or preconcentrate samples using magnetic markers. Using a simple 1D paper microfluidic device, researchers performed DNA extraction and loop-mediated isothermal amplification (LAMP), and detected DNA amplicons using lateral flow and antibody-based detection [33]. This device has a section that multiplexes the LAMP amplification and performs it three times as well as a control test. First, the sample was pretreated with functionalized magnetic particles which bound to *Plasmodium falciparum* L-lactate dehydrogenase (PfLDH). The sample was then loaded onto the chip, and a magnetic lens was placed underneath. By applying a current to the lens, the researchers formed virtual channels that connected the sample input to an absorbent wick. The magnetically tagged analytes were then trapped in the magnetic field while the supernatant was absorbed, leaving a large concentration of tagged PfLDH [33]. Paper microfluidics have also been shown to be useful in simpler preconcentration methods. One such application was shown by Bahmla et al. who used a circular paper disc, capillary tubes, and inertial forces to turn the disc into a low-cost centrifuge [35].

Although these applications are low-cost and require very little training to operate, the need for pre-processing increases the complexity of their application in situ. Because of this, other groups have researched methods that do not require tagging analytes with markers, such as magnetic beads or fluorescence tags.

## 5. Mechanobiology

**Figure 3:**
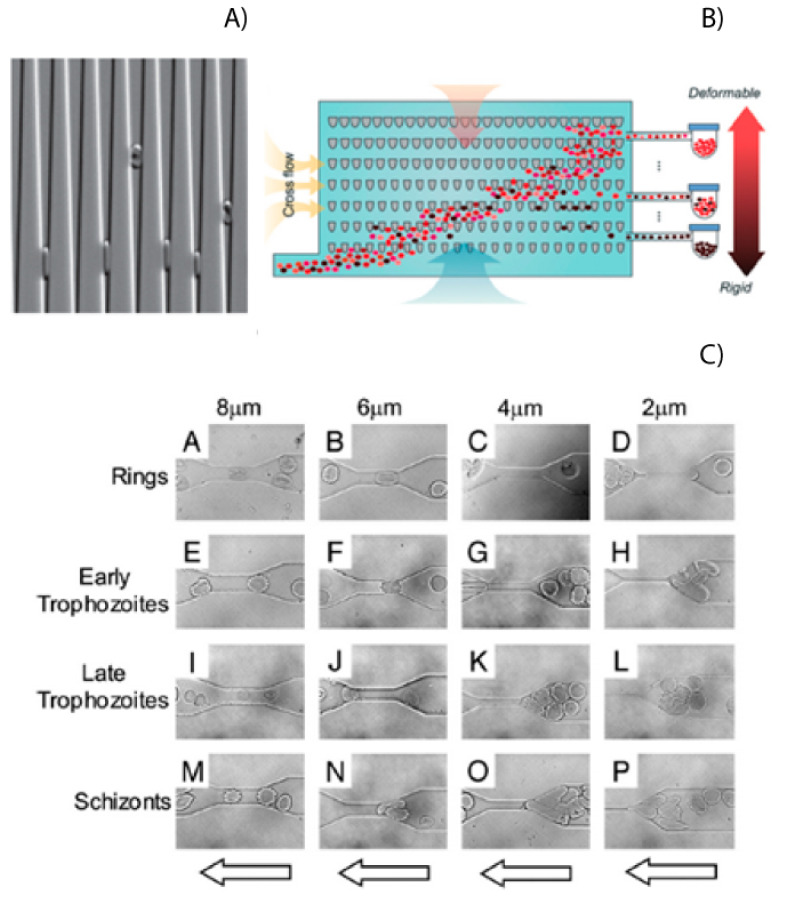
(**A**) RBCs and iRBCs in funnel shaped channels. The higher the deformability, the further the cells can travel downwards. Reprinted with permission from ref. [38] © 2009 Cellular Microbiology (**B**) Cell sorting by deformability using a matrix of funnel constrictions. Reprinted with permission from ref. [42] © 2016 The Royal Society of Chemistry (**C**) RBC traversing gaps in microchannels. Depending on the stage of the infection, the iRBC will become increasingly less deformable. Reprinted with permission from ref. [37] © 2003 National Academy of Sciences.

The rigidity of RBCs plays a significant role in the pathogenesis of malaria and can be used as a biomarker for malaria infection [36]. Microfluidics provides an excellent tool to study differences in cell physical–mechanical properties, such as the cell’s rigidity, size, and shape. Experiments have shown that infected red blood cells (iRBCs) could not traverse channels with width down to 2 or 4 μm, while healthy RBCs had no problems doing this due to their low rigidity (Figure 3A) [37,38]. Rigidity has also been shown as a possible biomarker for the stage that the parasite is in, as shown in Figure 3C. These properties of RBCs and iRBCs have been measures using microfluidic devices with wedges or a series of tapered constrictions [38,39]. The rigidity of iRBCs has also been investigated using a microfluidic device called a spleen-on-a-chip [40,41]. This study investigated how changes in cell rigidity caused more iRBCs to get stuck in the spleen.

This change in cell mechanics has also been used to separate healthy RBCs from iRBCS [41]. Using tapered constrictions and oscillatory flows, Guo could enrich samples of iRBCs up to 2500 times [42]. An illustration of this device is shown in Figure 3C. This reduced ability to deform has also been used to separate RBC and iRBCS in straight channels. The difference in rigidity will cause the iRCBs to migrate to the walls of a microfluidic channel, leaving the healthy RBCs in the center [43]. By using a three-outlet system, this margination can be used to separate the iRBCs from whole blood. Separations of up to 75% for early stage iRBCs and up to 90% for late stage iRBCs can be achieved.

Mechanobiological methods of cell separation are extremely promising due to their ability to their high level of efficiency. Unfortunately, the most effective method requires a complex setup and a low flow rate which dramatically limits the throughput. These limitations make it a poor candidate for use in remote locations. However, other methods exist that can also leverage the difference in rigidity and have much larger throughputs.

## 6. Inertial Microfluidics

In recent years, microfluidics has been gaining momentum as a viable method to pre-process biological samples using inertial forces. This method can be used to preconcentrate iRBCs, resulting in better diagnostics of malaria. Although only a few studies have been performed with malaria-infected RBCs, many have researched similar effects in separating CTC cells from whole blood samples. This application follows the same principle as the separation of iRBCs.

A large amount of research has gone into understanding how the forces in microchannels change with different channel cross sectional areas and shapes [42,44,45,46]. It uses the forces created by flowing a liquid through a channel at intermediate Reynolds numbers 1 < *Re* < 100. The inertial forces cause particles to migrate from one side of the channel to the other. The distance at which they focus depends on the size of the particle or their rigidity. For this to occur, the system must fulfill two criteria: (i) particle dimensions must be comparable to the characteristic length of the channel; and (ii) there needs to be a change in fluid velocity as you move from one wall of the channel to the other. These criteria create an asymmetry in the forces acting on the particle, causing it to migrate horizontally in the channel.

Studies have shown that at least two forces act on a particle in a straight channel. The shear gradient lift force $F_{sl}$ and the wall-induced lift force $F_{wl}$ [47,48,49]. The shear gradient lift force is created by the parabolic nature of the Poiseuille flow; this force pushes particles towards the walls of the channel. As the particle gets closer to the wall, a pressure imbalance is created, resulting in a force pushing away from the wall. For neutrally buoyant particles, other forces are negligible and the migration of particles are only affected by the $F_{sl}\;$ and $F_{wl}$ [50].

Secondary flows can be created by changing the channel’s curvature or by creating asymmetry in the channel. By adding curvature to the channel, a pressure gradient is created between the convex and concave walls. This gradient creates two counter rotating streams called dean vortices. Using these vortices and the interactions with the inertial lift force makes it possible to change the equilibrium position for particles in the channel. These positions depend on the physical properties of the particles and the forces acting on them.

**Figure 4:**
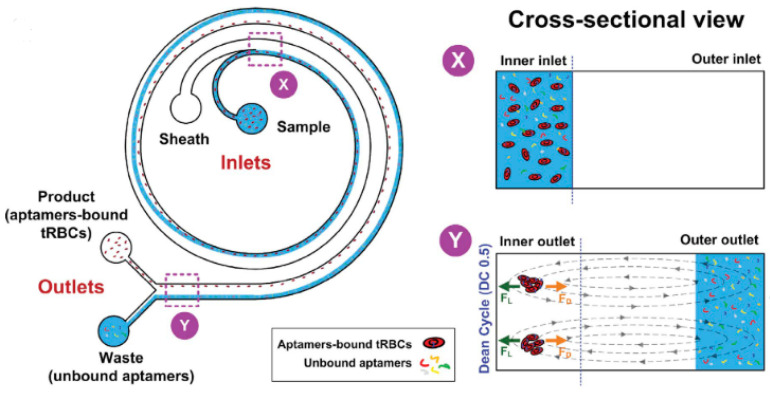
Results from inertial separation of unbound aptamers. Due to the dean drag forces, the unbound aptamers migrate towards the outer wall and into the water outlet. Bound aptamers and microfluidic beads experience a lift force which keeps them close to the inner wall. Reprinted with permission from ref. [51] © 2015 Scientific Reports.

Despite the successes of these applications in CTC diagnosis, the application of inertial microfluidics to other diseases is just beginning to be explored. Given that this method allows for the separation of cells using various biomarkers, such as size, shape, and rigidity, it has been proven to be a worthwhile field of study. So far, inertial microfluidics have only been used to separate unbound aptamers from those bound to infected red blood cells [51]. A diagram of the device and how it functions is shown Figure 4. However, preliminary evidence shows that this same method can be used to separate infected blood cells from healthy blood cells without the need for expensive equipment or special training.

## 7. Simulations

Computer simulations can give a deeper understanding of the effect of flow on particles and, thus, can help with the design of the microfluidic channel. The parameters that affect the particles in a microfluidic system include channel dimensions, flow rate, and the channel’s geometry. There is a wide range of methods for simulating microfluidics using a variety of software. One of the more popular and used software is COMSOL Multiphysics version 5.5 (COMSOL, Boston, MA, USA) with the microfluidics add-on module [52,53,54,55].

Martel et al. used COMSOL to calculate the equilibrium position of particles in straight and curved channels. It has been experimentally shown that particles suspended in a flowing liquid migrate across streamlines and reach an equilibrium position where they remain [56]. A study by Han Wei Hou et al. simulated the path of circulating tumor cells (CTCs), as well as red and white blood cells (RBC and WBC, respectively), in a spiral microchannel with a rectangular cross-sectional area [57]. In this research, the authors used the secondary dean flow to push particles of a specific size from the outer to the inner walls of the channel. COMSOL was used to study the dean flow at different input flow rates. They found that the positions of the streamlines were in good agreement with the experimental results and were able to use the computer model to aid in their microfluidic chip design. Research has also been carried out on other methods of cell separation, such as separation by cell rigidity [53]. Regarding studies on malaria, there are, again, only a few examples that were implemented in COMSOL. Simulations have mainly been used to test critical aspects of the designs, such as resistance/size of channels [42,58], fluid velocity, and particle trajectories [59], and strength of electric/magnetic fields [60,61] used in the discrimination between healthy and infected cells. Hsu et al. studied how to separate healthy avian RBCs from malaria-infected ones. They proposed that, by creating a device with a controlled shear rate, it was possible to use the morphological changes produced by malaria infections to separate healthy RBCs from malaria-infected ones [58]. The shear rates of the different designs were estimated using COMSOL. Simulations have also been used to help design a ratchet-sorting device [42]. Here, RBCs and infected red blood cells flow through a matrix of funnels with increasingly smaller gaps; more deformable cells will move further down the matrix. COMSOL was used to calculate the resistance of the individual funnels in the matrix and reduce the amount of trial and error involved in the final design. Another widespread use of COMSOL in microfluidics is in the calculation of velocity profiles and particle trajectories. Sivaraj et al. used COMSOL to develop a microfluidic chip that used microwear-based separation to isolate RBCs [59].

## 8. Microfluidic Impedance Flow Cytometry (MIFC)

Single-cell analysis is challenging; it requires tools and systems to manipulate and characterize cells by performing tasks, such as focusing, sorting, ordering, compressing, etc. [59]. As we have seen before, the stiffness of a cell can be used as a mechanical biomarker [36], but not only the mechanical properties can be used for this purpose. Another way to characterize individual cells is based on their electrical properties, such as impedance. This approach is called microfluidic impedance flow cytometry (MIFC), a microfluidic system that incorporates a pair of microelectrodes (or an array of them). Analyzing cells based on their impedance at different frequencies can provide important information about their physical and biological properties.

Electrical impedance represents the opposition of current flow through a given material or component and is defined as the ratio between an applied voltage and the resulting current. In the case of a MIFC, the component is a cell in a suspension medium. When an alternating voltage is applied at different frequencies, a complex impedance can be measured and calculated as follows:

(1) $$Z_{sys}^{*}=\frac{V^{*}}{I^{*}}$$

where $Z^{*}\;$is electrical impedance (Ohm), $V^{*}\;$ is applied voltage (Volts), and $I^{*}\;$ is current (Amp) (the * superscript denotes complex numbers).

**Figure 5:**
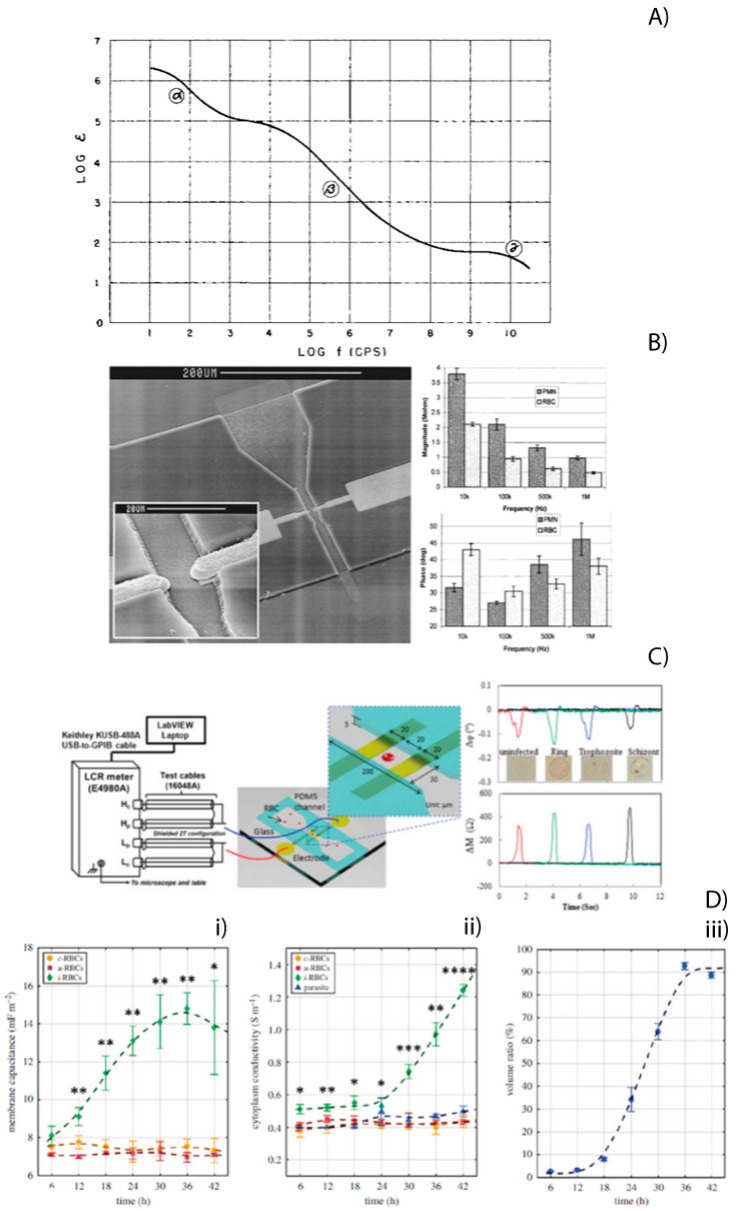
(**A**) The dielectric constant of muscular tissue as a function of frequency decreases in three major steps. The three steps are identified as a-, ß-, and 7-dispersion. Reprinted with permission from ref. [63] © 1957 ACADEMIC PRESS INC. Published by Elsevier Inc. (**B**) Electron scanning image of the channel and electrodes (Left). Magnitude and phase of the impedance values at different frequencies for PMN’s and RBC’s (Right). Reprinted with permission from ref. [66] © 1999 *IEEE Journal of Microelectromechanical Systems*. (**C**) Experimental MIFC system setup for Pf-iRBC detection using a microfluidic device (Left). EI transitions measured as uninfected RBCs and Pf-iRBCs crossed over the electrode probe. Measurement conditions: 2 MHz, 1 V, and 0.2% *w/v* BSA-PBS (Right). Reprinted with permission from ref. [69] © 2013 The Royal Society of Chemistry (**D**) Dielectric properties estimated using MMT modelling, during the parasite intraerythrocytic life cycle: (**i**) membrane capacitance, (**ii**) cytoplasm conductivity, and (**iii**) volume ratio occupied by parasites within the host cell. Mean values from the three TC experiments (symbols) and s.d. (error bars) are plotted for each time point. Smoothing splines (dashed lines) are plotted to represent the overall trend for each population. Statistical significance (Student’s *t*-test; * *p*, 0.05, ** *p*, 0.01, *** *p*, 0.001 and **** *p*, 0.0001) is represented for i-RBCs (N ¼ 3) versus c-RBCs and u-RBCs (N ¼ 6) at individual time points. Reprinted with permission from ref. [70] © 2018 Royal Society.

Already in 1956, Schwan, a pioneer in the field of bioimpedance analysis [62,63], used this method to measure the cell’s and tissue’s impedances in three main frequencies ranges: α dispersion (kHz), due to the double layer polarization around the cell [64]; β dispersion (MHz), which appears when the cell membranes begin to conduct current (capacitive effect); and γ dispersion (GHz), due to dipolar relaxation of water [65]. β dispersion is the most used region for microfluidic impedance flow cytometry. Figure 5A shows the dielectric constant of muscular tissue as a function of frequency.

Since Schwan, many developments and applications have been made based on bio-impedance analysis. Ayliffe et al. made the first microfluidic device with integrated microelectrodes for single-cell impedance measurements [66,67,68,69,70,71]. It consisted of a microchannel (4 × 10 ×150 μm) made of epoxy photoresist on glass and two gold electrodes (8 × 4 μm, with a gap of 7 μm). This device differentiated between two different cell types: human polymorphonuclear leukocytes (PMNs) and teleost fish RBCs. Figure 5B shows an electron scanning image of the device as well as magnitude and phase of the impedance values at different frequencies for PMNs and RBCs.

The malaria parasite’s infection of the RBC changes the physical–chemical structure of the host cell throughout a 48-h multi-stage life cycle. These changes also affect their electrical properties. It has been observed that the impedance of a healthy red blood cell is different from a plasmodium falciparum-infected red blood cell (pf-iRBC). Ribaut et al. [67] were the first to characterize pf-iRBC using MIFC, focused on the late intra-erythrocytic development stage. Abhai et al. [68] studied the progression of human cerebral malaria (CM) and its relationship to the invasion of pf-iRBC in the blood-brain barrier by using electrical impedance measurements. Du et al. [69] made considerable progress in differentiating each intraerithrocytic asexual stage of *Plasmodium falciparum* from a healthy RBC. The device had a measurement micro channel (30 × 200 × 5 μm) and two Ti/Au co-planar electrodes (10/100-nm thickness, 20-μm width, and 20-μm gap) connected to a precision LCR meter and computer. They also introduced a novel parameter to discriminate different disease states. Figure 3C illustrates the equipment used, the microelectrode configuration, and the magnitude and phase data obtained from the three parasite states of a pf-iRBC. Honrado C. et al. [70] used a microfluidic impedance cytometry chip with a fluorescence detection region to determine the dielectric properties of iRBCs at specific stages. They used an oblate spheroid model for early-stage pf-iRBCs and a spherical model for late-stage pf-iRBCs. Genetically modified parasites to express green fluorescent protein (GFP) were needed to perform the experiments. Figure 5d shows the estimated dielectric properties (membrane capacitance and cytoplasm conductivity) and volume ratio occupied by parasites within the host cell during the parasite intraerythrocytic life cycle. A weakness of the MIFC technique is the strong positional dependence of the cell in relation to the microelectrodes and the electric field generated between them. For optimal data collection, it is required to form a unicellular train of cells to interact with the electric field at the same position. To achieve this, one of the most used techniques is 3D hydrodynamic focus, which consists of aligning the cells by pushing them with additional fluids in the x, y (2D), and Z (3D) directions. Greater detail on this topic can be found in the work of Daguerre et al. [71]. As such, complex impedance is an exciting and valuable electrical biomarker. However, more studies are required on the change in the electrical properties of cells infected with *Plasmodium vivax*, i.e., the dominant species in countries such as Peru.

## 9. Conclusions

In this review, we have presented several microfluidic methods that have been shown to be suitable means of either pre-processing blood or detecting different pathogens present in a sample. A key advantage is that these methods are label-free; this eliminates the need for expensive reagents that may require specialized equipment and training. We presented four microfluidic methods: paper microfluidics, mechanobiological microfluidics, inertial separation, and impedance analysis. These methods can be sub-divided into methods for pre-processing (inertial, mechanobiological, paper with virtual channels) and diagnostics (paper and impedance). Mechanobiological methods are highly efficient at separating particles of different sizes and rigidities. The main disadvantage is that high enrichment rates can only be achieved at low flow rates and require an extra oscillatory flow. These flows need to be tuned correctly to achieve maximum efficiency. Although inertial separation and simpler forms of mechanobiological separation achieve much lower enrichment levels, they can be run at much higher flow rates and can speed up diagnostics. Some applications of inertial separation have been able to utilize only one fluid flow and thus simplify the operation of the final device. Using inertial separation as a means of pre-processing will increase the efficacy of the following diagnostic methods. This increase in efficacy allows POC technicians to use a wider array of tools, such as impedance analysis, which were previously unfeasible due to their low throughput or inability to identify low percentages of parasites without costly pre-processing. Recent advances in MIFC analysis have shown that it is possible to identify different stages of the parasite. These positive results, along with some of the difficulties associated with other diagnostics methods, such as PCR and RDT, make the combination of a low-cost and high-throughput pre-processing stage and a reliable and easy-to-use diagnostics stage a considerable step forward in the treatment of malaria.

Although these technologies have many positive aspects, some limitations need to be addressed before using POCs in remote locations. The first limitation is the fabrication method that is typically used in making microfluidic devices. The literature shows that the majority of researchers have used photolithography as a means of fabricating molds for microfluidic chips. More recently, advances in 3D printing have also made it possible to print molds. Even with these advances in fabrication, these methods are still prohibitively expensive for lower income countries. Given that these countries are most likely to suffer from malaria, it is vital to research new and cheaper fabrication methods that can be deployed in the countries that need them. Another limitation for all microfluidic methods, except paper microfluidics, is the need for a pump to push the liquid through the system. These pumps are often large and require maintenance to perform consistently. The best pumps for microfluidics, i.e., pressure pumps, are expensive and difficult to obtain in remote areas.

Even with these limitations, microfluidic separation and diagnostic methods provide an excellent way of reaching underserved communities. By utilizing microfluidic separation, it is possible to selectively remove cells of different sizes, shapes, and rigidities depending on the velocity and microfluidic effect that is being leveraged. A health care worker could then screen preconcentrated samples without any special training. A more reliable and straightforward diagnostic method would significantly reduce the mortality rates associated with infectious diseases such as malaria.

## Data Availability

Not applicable.
